# Temporomandibular Disorders and Headache: A Retrospective Analysis of 1198 Patients

**DOI:** 10.1155/2017/3203027

**Published:** 2017-03-21

**Authors:** Carlo Di Paolo, Anna D'Urso, Piero Papi, Francesco Di Sabato, Daniele Rosella, Giorgio Pompa, Antonella Polimeni

**Affiliations:** ^1^Department of Oral and Maxillofacial Sciences, “Sapienza” University of Rome, Rome, Italy; ^2^Department of Clinical Medicine, Headache Center, “Sapienza” University of Rome, Rome, Italy

## Abstract

*Aim*. Headache is one of the most common diseases associated with Temporomandibular Disorders (TMDs). The aim of this study was to evaluate, retrospectively, if headache influences TMD's symptoms.* Material and Methods*. A total sample of 1198 consecutive TMD patients was selected. After a neurological examination, a diagnosis of headache, according to the latest edition of the International Classification of Headache Disorders, was performed in 625 subjects. Patients were divided into two groups based on presence/absence of headache: Group with Headache (GwH) and Group without Headache (GwoH). Descriptive statistics and Chi-square index were performed.* Results*. Sociodemographic (gender, marital status, and occupation) and functional factors, occlusion (occlusal and skeletal classes, dental formula, and occlusal abnormalities), and familiar pain did not show a statistically significant correlation in either group. Intensity and frequency of neck pain, arthralgia of TMJ, and myalgia showed higher correlation values in GwH.* Conclusion*. This study is consistent with previous literature in showing a close relationship between headache and TMD. All data underlines that headache makes pain parameters more intense and frequent. Therefore, an early and multidisciplinary treatment of TMDs should be performed in order to avoid the overlay of painful events that could result in pain chronicity.

## 1. Introduction

Temporomandibular Disorders (TMDs) involve alterations of the temporomandibular joint (TMJ), masticatory muscles, and related structures. Many of the clinical and instrumental aspects of these disorders overlap with other medical disciplines like otology, neurology, psychiatry, and others [1]. Headache is one of the most common painful conditions; few people are spared during their lifetime by at least one episode of headache: it is estimated that about 90% of the general population in a year suffer from at least a headache episode [2]. Temporomandibular Disorders (TMDs) and headache are closely related pathologies; prevalence of headache in the dysfunctional population varies between 48% and 77%, while in the general population the prevalence of headache is around 45% [3–6]. Primary headaches as Migraine, ETTH (Episodic Tension Type Headaches), and CDH (chronic daily headaches) are more common in patients with TMD symptoms compared to individuals without headache [7]. According to several studies, there is a strong correlation between headache and other dysfunctional symptoms, such as joint noise, pain during mandibular movement, pain in the temporomandibular area, depression, anxiety, and poor sleep quality [8].

Patients with headache and TMDs reported significantly higher levels of pain and disability compared to patients with only TMDs [9, 10].

The aim of this study was to evaluate if headache influences TMD's symptoms; to investigate the research purpose two homogeneous groups of TMD patients with and without headache were analyzed.

## 2. Materials and Methods

### 2.1. Study Design

To address the research purpose, the authors designed and implemented a retrospective cohort study, conducted at the Department of Oral and Maxillofacial Sciences, at “Sapienza” University of Rome, and approved by the institution review board (ref. number 2086/15).

The study sample was composed of a population derived from patients presenting at the University's Department for TMDs management in an interval of time between January 2011 and December 2013, according to the inclusion and exclusion criteria.

Subjects eligible for study inclusion had TMD diagnosis, based on Diagnostic Criteria for Temporomandibular Disorders (DC/TMD) [11], and provided signed informed consent according to the World Medical Association's Declaration of Helsinki.

Patients were excluded from enrollment to the study, if they had an uncontrolled systemic disease, missing medical records, a history of mental disorders, or refused to enroll to this study.

Subjects with coexisting/history of drug administration, physiotherapy, and splint therapy for previous headache treatment were excluded from the study.

### 2.2. Headache Assessment

A total sample of 1198 TMD patients was selected. Presence of headache was analyzed using both clinical parameters recorded on patient's medical charts and answers given by patient on the DC/TMD Symptom Questionnaire.

Headache was found in 894 (75%) patients while in 304 (25%) was excluded.

In order to differentiate headache and perform a correct diagnosis to exclude false positives, all patients positive for headache were invited to undergo a neurological visit with a neurologist specialized in the diagnosis of primary headache according to the latest edition of the International Classification of Headache Disorders (ICDH-III) [12].

After the neurological visit, in accord with ICHD-III, a diagnosis of headache was performed in 625 patients.

Two hundred and sixty-nine patients were excluded from the study: in particular, 191 subjects did not undergo the neurological examination and 78 were found to be affected from other neurological diseases, such as atypical facial pain and cranial neuralgia.

### 2.3. Study Variables

The total sample of TMD patients composed by 929 (78% of initial TMD sample) was divided into two groups based on presence/absence of headache in TMDs:

1. Group with Headache (GwH), composed of *n* = 625 (67.3% of TMD sample).
2. Group without Headache (GwoH), composed of *n* = 304 (32.7% of TMD sample).

Based on the analysis of the medical charts, five categories of variables were considered:

1. Sociodemographic factors (gender, age, marital status, and occupation).
2. Types of pain (arthralgia, muscle pain, headache, familiar pain, neck pain, and emotional strain).
3. Functional aspects (maximum spontaneous mouth opening, expressed in mm).
4. Occlusion (occlusal and skeletal class, dental formula, occlusal abnormalities, and parafunctions).
5. Diagnostics (muscle pain, myofascial pain, myofascial pain with referral, TMJ pain, Disc Displacement (DD) with Reduction (R), Subluxation, DD with Reduction (R) and intermittent Mouth Opening Limitation (MOL), DD without R with MOL, DD without R without MOL, and degenerative joint disease).

Age was evaluated through a qualitative scale with the following subdivisions: 0–15 childhood, 16–25 adolescents/young adults, 26–40 adults, 41–50 middle-aged adults, 51–60 mature/older adults, 61–70 seniors, and 71+ elders.

The type of pain was evaluated for each individual patient, considering anatomical position and intensity.

Pain intensity (cephalic, joint, muscle, and cervical pain) was evaluated utilizing the Verbal Numeric Scale (VNS) [13], which uses numeric values (0–100) to decipher the intensity of pain, with the following division into classes of pain intensity: 0 (no pain); 0–20 (slight and episodic pain); 20–50 (moderate pain); 50–80 (severe pain); and 80–100 (very severe pain).

Familiar pain was identified as the pain that prompted the patient to the gnathological visit.

Emotional strain was assessed using the DC self-evaluation questionnaire system and investigating the presence/absence of stress time beyond the norm during the previous six months.

Maximum spontaneous mouth opening was evaluated using numeric values in millimeters (mm) according to the parameters set by the DC/TMD; then the value obtained for each patient was converted into a qualitative functional measurement, using the following division into classes: 0–20 (severe restriction), 21–30 (limited), 31–40 (mild limitation), 41–50 (normal), and 50+ (laxity).

Presence/absence of parafunctional habits was assessed through the patient's medical history and a clinical evaluation of the morphological-functional state of the skeletal muscles and of the dental-periodontal tissues.

### 2.4. Statistical Analysis

The prevalence of headache in patients with TMDs was analyzed proportionally among subjects from the Total Dysfunctional Sample (TDS) who tested positive to cephalic pain for the entire period considered (2011–2013). Confidence intervals were set at 95% to obtain a precise estimate of headache prevalence in the sample. The Chi-square index was performed to evaluate the significance of statistical correlation among the variables considered, with a level of statistical significance *α* = 0.05.

## 3. Results

Sociodemographic (gender, marital status, and occupation) and functional factors, occlusion (occlusal and skeletal classes, dental formula, and occlusal abnormalities), and familiar pain did not show a statistically significant correlation in either group.

**Figure 1:**
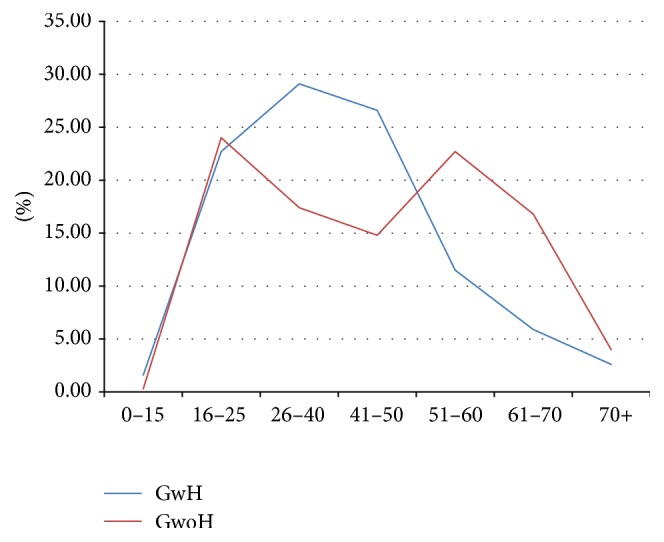
Line chart of frequency distribution for age intervals in Group with Headache (GwH) and Group without Headache (GwoH).

**Figure 2:**
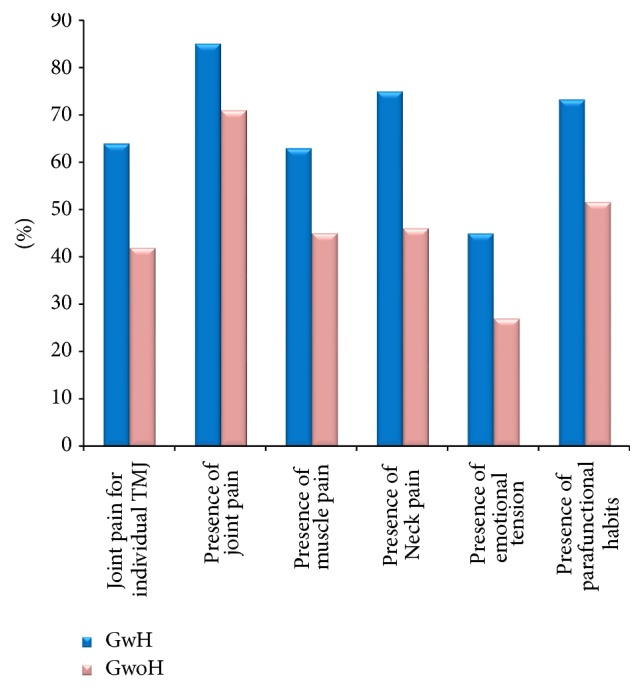
Percentage (%) values of prevalence of painful variables (joint pain for individual TMJ, presence of joint pain, muscle pain, and neck pain), emotional tension in the previous 6 months, and parafunctional habits in Group with Headache (GwH) and Group without Headache (GwoH).

In the TMD sample, headache prevalence was found to be 67.3% (*N* = 625, CI_95%_: 64.3%–70.3%). In GwH, pain score was 70 ± 24.4 VNS and 78% of patients (*N* = 487, Cl_95%_: 74.6%–81.4%) showed VNS values higher than 50. According to the ICHD-III, the types of headache diagnosed by the neurologist were in order of decreasing frequency: Migraine without Aura (MwoA), Episodic Tension Type Headache (ETTH), Chronic Migraine (CM), Chronic Tension Type Headache (CTTH), Migraine with Aura (MwA), Headache attributed to TMD (TMD Headache), and Headache attributed to disorder of Neck (Neck Headache) (Table 1). Statistically significant differences were observed in age classes between GwH and GwoH (Table 2) and in their graphic trends (Figure 1). In GwH, the age classes most frequently involved were 26–40 (*N* = 182) and 41–50 (*N* = 166). Patients of 16–25 (*N* = 73) and 51–60 (*N* = 69) were the most represented in GwoH. All variables considered showed higher values in GwH, while absence of pain was more frequent in GwoH (Table 3; Figure 2).

The statistical analysis demonstrated a significant correlation between presence of joint pain and headache (*X*-squared value = 24.3216) and between intensity of same-side joint and cephalic pain (*X*-squared value = 59.1496). The following associations were found to be statistically significant: intensity of muscular pain and ipsilateral headache; neck pain and headache (Table 4). Emotional strain (GwH = 45%, CI_95%_: 41.1%–48.9%; GwoH = 27%, Cl_95%_: 22.1%–31.9%) and parafunctional habits (GwH = 73%, CI_95%_: 69.6%–76.4%; GwoH = 52% CI_95%_: 46.4%–57.6%) showed higher values in GwH, with a correlation between headache and parafunctions (*X*-squared values = 42.7842) (Table 4; Figure 2).

DC/TMD pathologies such as myalgia, arthralgia, and reducible disc dislocation were found to be more frequent in GwH. TMJ pain, Disc Displacement with Reduction, and myofascial pain showed a correlation between the side of pathology and ipsilateral headache, although with lower correlation coefficients. Instead, DD with Reduction and intermittent Mouth Opening Limitation were more common in GwoH, although with lower correlation coefficients (*X*2 value = 5.1456, df = 1, *χ*_1,0.95_^2^ = 3.84). Other diseases demonstrated lower or insignificant correlation (Table 5).

## 4. Discussion

Headache appeared to be the most common symptom of diseases accompanying TMD [13]. Ciancaglini and Radaelli [14] suggested that 70% of headache patients had also a clinical confirmation of TMD. On the other hand, TMDs were also prevalent in subjects with headache [15, 16]. In the population of our study, headache prevalence was found to be of 67.3% (Cl_95%_: 64.3%–70.3%), in agreement with current literature.

Other studies [16–18] showed how primary headaches are more frequent in the dysfunctional patients compared to the control group. According to our findings, MwoA and ETTH were the most frequently reported primary headaches associated with TMD patients, with a prevalence of 29.9% (*N* = 187, Cl_95%_: 26.3%–3.5%) and 24.5% (*N* = 153, Cl_95%_: 21.1%–27.9%), respectively.

In the dysfunctional Group with Headache (GwH), the peak age group was “30–40” (Figure 1). These results are in agreement with another study on headache and TMD that showed a higher prevalence between 20 and 40 years of age with a subsequent tendency to decrease [19]. On the other hand, two higher peaks were found in GwoH: one between 16 and 25 yrs and another one in the 40–60-year group (Figure 1).

The different behavior between GwH and GwoH could be explained by the fact that headache determines an increased central sensitization to pain and an exacerbation of pain symptoms in the craniocervical-mandibular joint, thus motivating patients to seek treatment for previously existing gnathologic problems [20].

Central sensitization is characterized by pain hypersensitivity, particularly dynamic tactile allodynia, secondary punctate or pressure hyperalgesia, aftersensations, and enhanced temporal summation [20, 21].

In addition, absence of severely painful symptoms might induce patients to underestimate dysfunctional problems, which could become discernible only in the chronic phase, explaining the second age peak (40–60 years) in GwoH.

Several studies [22, 23] showed a correlation between frequency and intensity of headaches and presence of TMDs. Patients with headache and TMDs reported statistically significant higher levels of pain and disability compared to patients with only TMDs [10]. This may explain why headache was found to be more frequent and intense in dysfunctional patients compared to the control population [24]. Furthermore, debilitating headache (VNS score > 50) was found in the majority (78% Cl_95%_: 75%–81%) of dysfunctional patients in our study. These results suggest that when patients report a severe headache, a clinical assessment of the morphofunctional state of temporomandibular joints and masticatory muscles should be performed in order to exclude the presence of TMDs. Our research found that painful pathologies of the craniofacial region are more frequent and intense in patients who suffer simultaneously from headache. Compared to GwoH, the increase in pain in GwH could also be explained by the greater central and peripheral nervous sensitivity that characterizes patients with headache. In dysfunctional patients, there is a significant association between presence of painful sites during palpation in areas innervated by the trigeminal nerve and headache frequency [25–27]. According to the pain adaptation theory [28], different types of pain tend to reinforce each other. In support of this hypothesis, data showed a direct proportionality between intensity of pain and headache (Table 3). This finding is also confirmed by analysis of pathologies considering the side of the body involved: when dysfunctional pain (joint; muscle) and/or cervical pain was located on the same side of headache, pain tended to be directly proportional to headache (Table 4). In GwH, the kind of pain most frequently associated with headache, regarding frequency, intensity, and statistical correlation, was found to be neck pain (Tables 3 and 4). Some researchers have evaluated the relationship between cervical stimulation and onset of headache, in particular the presence of cervical trigger points ipsilateral to headache site [29, 30]. This phenomenon is explained by the presence of cervical nerve afferents in the trigeminal nerve nuclei [31].

Emotional tension and parafunctions were more frequent in GwH compared to GwoH, respectively, 18.1% (Cl_95%_: 12.3%–23.9%) and 21.7% (Cl_95%_: 19.7%–23.7%) (Figure 2). A correlation was also found between the presence of parafunctions and headache (*x*-square values = 42.7842) (Table 4). Independent of mechanism and direction of the association between parafunctions and headache, our data showed similar results of other studies that have underlined how the presence of parafunctions may favour headache presence and vice versa [32]. In the two groups analyzed, there was an even distribution of DC/TMD diseases, with the most frequent being joint pain, which was present in 70–80% of patients. In TMD patients, a higher prevalence of joint problems than muscular problems was found (Table 5). Data that emerged in this study showed that headache has a different relationship with muscle pain compared to joint pain.

According to our findings, headache was found to be related to muscle pain with respect to frequency, while it is found to be associated with joint pain with respect to intensity (*x*-square = 27.4131 versus *x*-square values = 59.1496) (Table 4). In particular, joint pain seems to exacerbate headache more than muscle pain. Finally, it can be assumed that in a dysfunctional patient the reduction of headache intensity and frequency can be achieved by the improvement of joint and muscle function, respectively.

## 5. Conclusion

This study is consistent with previous literature in showing a close relationship between headache and TMD. Data underlines that headache makes pain parameters more intense and frequent, complicating dysfunctional diseases both in the diagnostic phase and in treatment. Therefore, it is desirable to perform an early and multidisciplinary treatment of TMDs in order to avoid the overlay of painful events that could result in pain chronicity.

## Figures and Tables

**Table 1:** Absolute frequency (*N*) and percentage (%) values of headache types in Group with Headache (GwH), according to the ICHD-III. ETTH: Episodic Tension Type Headache; CTTH: Chronic Tension Type Headache; MwA: Migraine with Aura; MwoA: Migraine without Aura; CM: Chronic Migraine; TMD Headache: Headache attributed to TMD; Neck Headache: Headache attributed to disorder of Neck.

ICHD-III diagnosis	*N* (%)	Cl_95%_
ETTH	153 (24.5%)	Cl_95%_: 21.1%–27.9%
CTTH	54 (8.6%)	Cl_95%_: 6.6%–10.6%
MwA	51 (8.2%)	Cl_95%_: 6.1%–10.3%
MwoA	187 (29.9%)	Cl_95%_: 26.3–3.5%
CM	136 (21.8%)	Cl_95%_: 18.6%–25.0%
TMD Headache	32 (5.1%)	Cl_95%_: 3.4%–6.8%
Neck Headache	12 (1.9%)	Cl_95%_: 0.9%–2.9%

**Table 2:** Absolute frequency (*N*) and percentage (%) values of age intervals in Total Dysfunctional Sample (TDS) and in Group with Headache (GwH) and Group without Headache (GwoH).

Age	TMD sample		GwH		GwoH	
	*N* (%)	Cl_95%_	*N* (%)	Cl_95%_	*N* (%)	Cl_95%_
0–15	11 (1.2%)	Cl_95%_: 0.5%–1.9%	10 (1.6%)	Cl_95%_: 0.6%–2.6%	1 (0.3%)	Cl_95%_: 0.6%–2.6%
16–25	215 (23.1%)	Cl_95%_: 20.7%–25.8%	142 (22.7%)	Cl_95%_: 19.7%–25.7%	73 (24.0%)	Cl_95%_: 0.6%–2.6%
26–40	235 (25.3%)	Cl_95%_: 22.5%–27.9%	182 (29.1%)	Cl_95%_: 26.1%–32.1%	53 (17.4%)	Cl_95%_: 0.6%–2.6%
41–50	211 (22.7%)	Cl_95%_: 20.1%–25.3%	166 (26.6%)	Cl_95%_: 23.6%–29.6%	45 (14.8%)	Cl_95%_: 0.6%–2.6%
51–60	141 (15.2%)	Cl_95%_: 19.9%–17.5%	72 (11.5%)	Cl_95%_: 9.5%–13.5%	69 (22.7%)	Cl_95%_: 0.6%–2.6%
61–70	88 (9.5%)	Cl_95%_: 7.7%–11.3%	37 (5.9%)	Cl_95%_: 4.1%–7.7%	51 (16.8%)	Cl_95%_: 0.6%–2.6%
70+	28 (3.0%)	Cl_95%_: 2.0%–4.0%	6 (2.6%)	Cl_95%_: 1.4%–3.8%	12 (4.0%)	Cl_95%_: 0.6%–2.6%

**Table 3:** Absolute frequency (*N*) and percentage (%) values of prevalence of painful variables of cranium-facial districts (presence of joint pain, muscle pain, and neck pain) in Group with Headache (GwH) and Group without Headache (GwoH). TMJ: temporomandibular joint. VNS: Verbal Numeric Scale (0–100).

VNS	TMJ pain			
	GwH		GwoH	
	*N* (%)	Cl_95%_	*N* (%)	Cl_95%_
0	93 (14.9%)	Cl_95%_: 12.2%–17.6%	89 (29.3%)	Cl_95%_: 24.2%–34.4%
1–20	76 (12.2%)	Cl_95%_: 14.7%-14.7%	42 (13.8%)	Cl_95%_: 10%–17.6%
21–50	112 (17.9%)	Cl_95%_: 14.9%–20.9%	57 (18.8%)	Cl_95%_: 14.4%–23.2%
51–80	234 (37.4%)	Cl_95%_: 33.6%–41.7%	80 (26.3%)	Cl_95%_: 21.4%–31.2%
81–100	110 (17.6%)	Cl_95%_: 4.6%–20.6%	36 (11.8%)	Cl_95%_: 8.2%–15.4%

VNS	Muscle pain			
	GwH		GwoH	
	*N* (%)	Cl_95%_	*N* (%)	Cl_95%_

0	231 (37%)	Cl_95%_: 33.2%–40.8%	166 (54.6%)	Cl_95%_: 49.0%–60.2%
1–20	45 (7.2%)	Cl_95%_: 5.2%–9.2%	26 (14.8%)	Cl_95%_: 10.9%–18.7%
21–50	120 (19.2%)	Cl_95%_: 16.1%–22.3%	45 (8.6%)	Cl_95%_: 5.5%–11.7%
51–80	51 (8.2%)	Cl_95%_: 6.1%–10.3%	22 (14.8%)	Cl_95%_: 10.9%–18.7%
81–100	178 (28.4%)	Cl_95%_: 24.9%–31.9%	45 (7.2%)	Cl_95%_: 4.3%–10.1%

VNS	Neck pain			
	GwH		GwoH	
	*N* (%)	Cl_95%_	*N* (%)	Cl_95%_

0	159 (25%)	Cl_95%_: 21.7%–28.3%	160 (52.6%)	Cl_95%_: 47%–58.2%
1–20	39 (6%)	Cl_95%_: 4.1%–7.9%	14 (4.6%)	Cl_95%_: 2.3%–6.9%
21–50	85 (14%)	Cl_95%_: 11.3%–16.7%	33 (10.9%)	Cl_95%_: 7.4%–14.4%
51–80	169 (27%)	Cl_95%_: 23.5%–30.5%	67 (22%)	Cl_95%_: 17.4%–26.6%
81–100	173 (28%)	Cl_95%_: 24.5%–31.5%	30 (9.9%)	Cl_95%_: 6.6%–13.3%

VNS	Headache			
	GwH			
	*N* (%)		Cl_95%_	

1–20	50 (8%)		Cl_95%_: 5.9%–10.1%	
21–50	88 (14%)		Cl_95%_: 11.3%–16.7%	
51–80	231 (37%)		Cl_95%_: 33.2%–40.8%	
81–100	256 (41%)		Cl_95%_: 37.2%–44.8%	

**Table 4:** *X*-squared value of correlation between presence and intensity of headache and presence and intensity of muscle, joint, and/or cervical ipsilateral pain and between presence of headache and presence of parafunctional habits.

	*X*-squared presence	*X*-squared intensity
Ipsilateral headache and joint pain	24.3216	59.1496
	*χ*_1,0.95_^2^ = 3.84	*χ*_9,0.95_^2^ = 16.92
Ipsilateral headache and muscle pain	22.1273	27.4131
	*χ*_1,0.95_^2^ = 3.84	*χ*_9,0.95_^2^ = 16.92
Ipsilateral headache and cervical pain	97.3326	81.3128
	*χ*_1,0.95_^2^ = 3.84	*χ*_9,0.95_^2^ = 16.92
Parafunctional habits	42.7842	—
	*χ*_1,0.95_^2^ = 3.84	

**Table 5:** Absolute frequency and percentage [*n* (%) values] of DC/TMD diseases in Group with Headache (GwH) and Group without Headache (GwoH); difference in prevalence [*n* (%) values]; and *X* squared value of correlation between presence of headache and dysfunctional diseases. DD with R: Disk Displacement with Reduction, MOL: Mouth Open limitation, and DD without R: Disk Displacement without Reduction.

DC/TMD disease	*GwH*		GwoH		Difference in prevalence in favour of *GwH* or GwoH		*X* ^2^ value of correlation between headache and dysfunction
	*N* (%)	Cl_95%_	*N* (%)	Cl_95%_	*N* (%)	Cl_95%_	
Muscle pain	395 (63%)	59.2%–66.8%	138 (45%)	39.4%–50.5%	*257 (18%)*	*15.7%–20.5%*	22.1273 *χ*_1,0.95_^2^ = 3.84
Myofascial pain	119 (20%)	6.9%–23.1%	42 (14%)	10.2–17.9%	*77 (6%)*	*4.5%–7.5%*	3.896 *χ*_1,0.95_^2^ = 3.84
Myofascial pain with referral	59 (9%)	6.1%–11.9%	20 (7%)	4.1%–9.9%	*39 (2%)*	*1.1%–2.9%*	—
TMJ pain	747 (85%)	82.2%–87.8%	251 (71%)	65.9%–76.1%	*496 (14%)*	*11.8%–16.2%*	24.3216 *χ*_1,0.95_^2^ = 3.84
DD with R	364 (58%)	54.1%–61.9%	144 (47%)	41.4%–52.6%	*220 (11%)*	*9%–13%*	14.7635 *χ*_1,0.95_^2^ = 3.84
Subluxation	91 (15%)	12.2%–17.8%	35 (12%)	8.4%–15.6%	*56 (3%)*	*2%–4%*	—
DD with R and intermittent MOL+	11 (2%)	1.1%–2.9%	13 (4%)	2.1%–5.9%	2 (2%)	1.1%–2.9%	5.1456 *χ*_1,0.95_^2^ = 3.84
DD without R with MOL	45 (8%)	5.9%–10.1%	29 (10%)	6.6%–13.4%	16 (2%)	1.1%–2.9%	—
DD without R without MOL	19 (3%)	1.7%–3.3%	9 (3%)	1.1%–4.5%	0 (0%)	—	—
Degenerative joint disease	48 (8%)	5.9%–10.1%	27 (9%)	5.8%–12.2%	21 (1%)	0.4%–1.6%	—
